# Crowd Agile Model for Effective Software Development

**DOI:** 10.1007/978-3-030-58858-8_28

**Published:** 2020-08-18

**Authors:** Shamaila Qayyum, Salma Imtiaz, Huma Hayyat Khan

**Affiliations:** 6 IT University of Copenhagen, Copenhagen, Denmark; 7 University of British Columbia, Vancouver, BC Canada; 8 International Islamic University, Islamabad, Pakistan; 9 National University of Modern Languages, Islamabad, Pakistan

**Keywords:** Agile, ASD, Scrum, Crowd source software development, CSSD

## Abstract

Crowd Sourced Software Development (CSSD) is becoming popular in software development industries due to reduced cost and efficiency. Many companies are moving towards crowdsourcing, and have already adopted Agile Software Development (ASD). However, CSSD differs from ASD in many ways due to its distributed nature. Although there is little research on the integration of these two approaches, whereas at the same time the combination of the two is advocated by some. It is deemed necessary to identify and resolve the issues emerged while integrating CSSD and ASD. This study hence intends to explore the issues emerged as a result of integrating agile and CSSD and propose a Crowd Agile model that will help in effective software development.

## Introduction

With the growth of software industry, traditional software development practices are getting old [1–3]. Internet and social media have provided ways for developers and employers to reach each other across the globe to get their task done [1, 3]. This approach of developing software which utilizes people around the globe for various kind of tasks is known as crowdsourcing [4, 5]. Recently Crowd Sourced Software Development (CSSD) has taken over the software industry [6]. The phenomenon involves outsourcing the tasks to the crowd consisting of unknown, heterogeneous people by an open call [4, 7]. The crowd, coordinates via any online platform [8] such as Amazon Mechanical Turk and Topcoder, to complete the tasks given by the employer [7]. This technique has said to improve the quality of tasks by increasing response time and reducing the cost [9].

CSSD enables the abilities of assorted individuals to be incorporated into a single venture [12]. It is commonly used for coding [13–16] and testing [17–23] in software development. In crowdsourcing, people performing tasks are heterogeneous gatherings, who do not know each other. As the software requirements are becoming unpredictable and researchers are emphasizing on more communication within team [24], CSSD is facing the challenges of team development [4, 9], developing volunteers’ network [8], task decomposition, trust among the crowd working on same project [14] and coordination.

On the other hand agile methodology is also a very popular software development methodology [25, 26] as it enables software teams to deliverable valuable services in flexible manner [27, 28]. It has been established that many companies adopting CSSD are also following ASD [29]. Companies, such as TPI, that use TopCoder as a platform, are shifting towards crowdsourcing and are already following agile methods [30]. The combination of crowd sourced software development and agile is one of an interesting and latest research area. As stated by Mishra [29], lately practitioners are following crowd source approach while working in agile environment, but research lacks sufficient articles on the effective integration of these two approaches. Stol [30] also highlighted the importance of effective combination of crowd and agile. The research is still very new and limited. Therefore, we aim to conduct an exploratory research to determine what are the challenges faced when CSSD is used within agile environment and what strategies can be followed to reduce these challenges, followed by an explanatory research in which we will validate our proposed model.

The rest of the document is divided into following sections; Sect. 2 gives the background on CSSD and agile. Section 3 provides literature review and recent state of art of agile and CSSD. Section 4 contains the objectives of this study and research questions this study intends to follow. Section 5 states detailed research methodology. Section 6 presents the contribution of this research and Sect. 7 provides a timeline in which this research will be completed.

## Background and Motivation

In the recent years a new trend of software development has become popular in industry that lets the organizations involve isolated group of developers to develop software projects [1–3]. This concept is known as crowdsourcing. The term crowdsourcing was initially defined by Howe [10] in 2006. Social networking is one reason behind this globalization [1, 3]. A crowd can be any group of large number of anonymous people, which may comprise of experts as well as fresh graduates and inexperienced people. Crowdsourcing helps utilizing the skills of different people to be integrated into a single project [12].

At the same time, companies are widely adopting agile software development methodology [33]. Interestingly, some of the characteristics that are faced as challenges by CSSD are the benefits achieved as a result of following ASD. Agile not only keeps the team productive and motivated, but provides quality products [34]. ASD emphasizes on face to face communication [35, 36] and works in iteration with close collaboration of team, project manager and business people [27, 37]. Trust development among team members is another strength of ASD [38]. A daily meeting is held to discuss the progress of the project [34, 39]. ASD also helps in lowering cost of project as there is increased communication which decreases the chances rework and therefore cost overrun and project delay [24]. However, it is problematic executing CSSD in an agile environment [33].

Agile Software Development (ASD) has been adopted by the organizations doing Global Software Development (GSD) [27, 40–43]. GSD leads software developing companies, develop the software across remotely located teams [44]. Since many practices of agile are thought to solve different challenges of the organizations working across the globe [44–46]. CSSD is different from GSD, in GSD there are designated teams and CSSD comprise of a large group of unknown people who do not know each other neither are they designated employees of the organization [33]. Since agile practices can benefit GSD [33] we suggest they can be integrated and used during CSSD. How both can be effectively integrated needs to be explored [4]. This motivated us to conduct a research on exploring how agile and CSSD can be used together in a way that the maximum benefits of both the approaches can be achieved.

## Related Work

### State of Knowledge on Crowd Sourced Agile Development

After reviewing recent literature we found a few studies on crowd sourced agile development. The most recent study among them on crowd source agile development was conducted by Klass Jan Stol [30] in 2019. They conducted interviews and found that the managers, who were involved in CSSD as well as agile, faced many problems because of these contradictory approaches. Researchers emphasized the need to investigate how these two approaches can be combined effectively. Likewise, another study by Klass Jan Stol is conducted in 2014 [4], where the researchers reported the significance of crowdsourcing. Mishra and colleagues in 2017 [29] conducted a study that reports the importance of integrating CSSD and ASD. The researchers highlighted the recent trends towards investigating agile development with crowd sourcing.

CSSD [1] and ASD [34] are widely adapted by the companies today since there are some key issues in following these both simultaneously as many characteristics of agile and crowdsourcing are contradicting each other. As [30] stated:

*“Given the widespread adoption of agile approaches to software development (in particular Scrum) that emphasize regular face*-*to*-*face communication, how can the crowdsourcing approach (which resembles a waterfall*-*style approach to software development with an emphasis on documented requirements) be effectively combined and coordinated?”*

Literature shows that ASD is also used by organizations doing GSD [40], [60]. Since GSD also has many characteristics that contradict with the principles of ASD. However, it has been established that agile practices are used for mitigating many challenges of GSD [44–46]. GSD differs from CSSD as in GSD there are designated teams but in CSSD a large group of unknown people is working on same task [33]. However, in CSSD the workers are globally dispersed as the designated teams in GSD are working from remote locations, so geographical difference is a commonality among both. If agile practices can be used for GSD, where teams are geographically apart from each other, it can also be used within CSSD for effective software development.

## Research Questions

The study aims to explore the challenges of CSSD when used with ASD and the solutions that help reduce these challenges. This motivated us to design our research questions, as follows:

### RQ1:

How can Crowd Sourced Software Development (CSSD) be effectively integrated with Agile Software Development (ASD) to achieve maximum benefits of both?

*RQ 1.1:* What are the challenges when ASD is used with CSSD?

*RQ 1.2:* What strategies could be used to overcome the challenges of ASD and CSSD integration?

RQ 1.3: How can the crowd agile model help CSSD teams be more successful in their use of ASD?

## Methodology

To answer each research question of the study, following research methodology will be adapted. Table 1 gives a detailed research summary along with the methodology.

**Table 1.** Research summary

Research question	Objective	Methodology	Outcome
RQ 1.1	To identify the challenges of integrating CSSD and ASD from literature as well as industry	- Literature review - Survey	List of issues faced by companies following crowd source approach with agile software development
RQ 1.2	To identify the practices that can address the identified challenges of CSSD and ASD	- Literature Review - Survey	- List of solution strategies adapted by companies to resolve issues while using crowd sourcing approach within agile environment
	To propose a Crowd Agile model		- a model based on the issues and solutions identified in previous phases
RQ 1.3	To evaluate how crowd agile model helps in effective software development	- Case study - Plan B - Experiment	An evaluated model

*Literature review (LR)* will be conducted in order to find the reported issues while working together with crowd sourcing and agile. LR will also be carried out to find the solutions and strategies for the issues emerged that are given in the literature.

A *survey* will be conducted to find out what issues practitioners are facing when they use crowdsourcing while working in an agile environment. This survey will also figure out what strategies these practitioners adopt to overcome the issues faced by combining crowdsourcing with agile. The survey will be *exploratory* as we will be identifying issues and the strategies that are faced by industry. Guidelines for conducting survey by Kasunic [47] will be followed. T*ype of survey:* This survey will be self-administered. Self-administered surveys are those which are sent online through web based surveys or emails. The survey will be a mix of both open and closed questions. *Target audience:* The targeted audience for our survey is the software development practitioners and managers working with crowdsourcing and agile. The target audience will be approached by different platforms which allow crowdsourcing. It will be first confirmed through email/any other contact medium that they are also following agile.

*Case study:* The evaluation of the model proposed in this study, will be done with a case study. For carrying out case study effectively, guidelines for conducting and reporting case study research in software engineering by Runeson [48] will be followed. Case study will be *explanatory*. This study intends to find how CSSD and agile can be integrated and what benefits can be achieved from this integration. *Case definition*: The case of any software development company will be considered who carry out software engineering activities through crowdsourcing while working in agile environment. *Unit of analysis:* Our unit of analysis will be the software project and the practitioners that will develop software using our model. *Data Collection:* Data collection from the case study will be done through second level technique. The researchers will directly collect raw data by monitoring the use of the model. This will be done using *interviews. Data analysis:* Qualitative data analysis will be done. Narrative analysis and discourse analysis will be used for this purpose. As the data will be received in the form of different responses from the respondents’’ so it will be better analyzed in the form of respondents’ stories, talks and texts.

We understand the issues in identifying an organization for case study, as a plan B, we will conduct an experiment for the evaluation of our proposed model.

## Research Framework

### Theoretical Perspective of Research:

The theoretical perspective of this study is based on organizational theory as it focuses on the improvement in organizational performance. A positivist approach is being followed as the research aims to study human behavior and results are produced on empirical basis.

### Methodological Perspective of Research:

This is *applied research* as the sole purpose of carrying out this research is to address the problems of an organization which is using ASD along with CSSD. The type of applied research this study follows is *evaluation research*, as we intend to study the impact of our suggested model.

## Expected Outcomes

The outcome of this study will be in the form of a model. In this model guidelines will be provided for effective use of CSSD with ASD. These guidelines will be in the form of challenge-strategy pair. This pair will specify that for any particular challenge (while integrating CSSD with ASD), what strategy can be followed so that the benefits of both can be achieved. The contribution of this research will be; the identification of issues while using CSSD with agile, the identification of solutions for the issues identified while using CSSD with agile, a crowd agile model for effective usage of CSSD and agile and validation of the model.
